# A refined two-step oligoribonucleotide interference-PCR method for precise discrimination of nucleotide differences

**DOI:** 10.1038/s41598-018-35479-0

**Published:** 2018-11-21

**Authors:** Toshitsugu Fujita, Miyuki Yuno, Fusako Kitaura, Hodaka Fujii

**Affiliations:** 1 Department of Biochemistry and Genome Biology, Hirosaki University Graduate School of Medicine, 5 Zaifu-cho, Hirosaki, 036-8562 Aomori Japan; 2 Chromatin Biochemistry Research Group, Combined Program on Microbiology and Immunology, Research Institute for Microbial Diseases, Osaka University, 3-1 Yamadaoka, Suita, 565-0871 Osaka Japan

## Abstract

We previously developed oligoribonucleotide (ORN) interference-PCR (ORNi-PCR), in which an ORN hybridises with a complementary DNA sequence and inhibits PCR amplification across the sequence in a sequence-specific manner. Suppression of target amplification by ORNi-PCR can be used to detect nucleotide differences such as mutations in a target sequence. In the present study, we refined the ORNi-PCR method and established a detailed technical protocol to precisely discriminate single-nucleotide differences. We first revealed that a two-step (denaturing and annealing plus elongation) rather than a standard three-step (denaturing, annealing and elongation) method is more suitable for stably hybridising an ORN to its target DNA sequence for sequence-specific suppression of target amplification. We then optimised the ORNi-PCR method using two-step cycles and established a step-by-step technical protocol. The optimised Two-Step ORNi-PCR method could discriminate single-nucleotide differences in genomic DNA or cDNA introduced by genome editing or mutations in cancer cells. In addition, we showed that Two-Step ORNi-PCR can detect the cancer cells possessing a single nucleotide mutation in a target locus mixed with a large number of cells harboring wild-type sequences in the locus so that the number of the cancer cells is only 0.2% of the total cell number. Two-Step ORNi-PCR is useful for simple, precise, cost-effective and positive detection of nucleotide differences in a wide range of molecular biology and medical applications.

## Introduction

PCR is an established method for amplifying nucleotides of interest that is widely used in various fields. Although PCR can specifically amplify target sequences, annealing of designed primers with non-target sites can result in non-specific amplification of non-target amplicons. In addition, PCR using a given primer set can amplify both intact and mutated DNA, making it difficult to selectively amplify only the target DNA. To avoid such undesirable amplification, various methods have been developed^1^. For example, blocking PCR can suppress non-specific amplification and be used for detection of specific DNA and discrimination of nucleotide mutations^1^. Blocking PCR utilises 3′-modified DNAs and artificial nucleic acids such as locked nucleic acids (LNAs) and peptide nucleic acids (PNAs), which are complementary to the target sequence and block elongation by DNA polymerase or compete with primers for annealing. Although such artificial nucleic acids may display higher stability, affinity for target DNA or resistance to nucleases, their synthesis is more expensive than that of 3′-modified DNAs.

**Figure 1:**
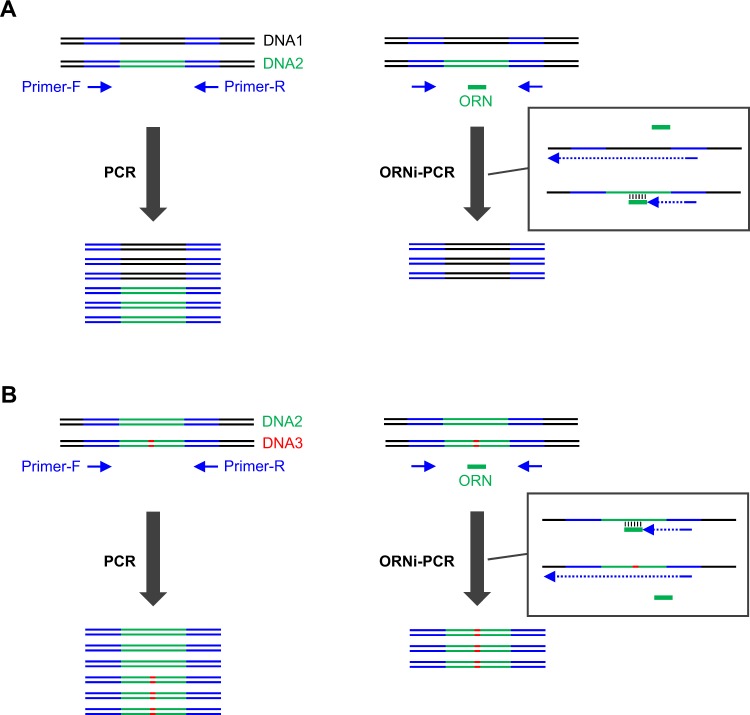
Schematic diagram of ORNi-PCR and its application to the detection of nucleotide differences. (**A**) An oligoribonucleotide (ORN), not an oligodeoxyribonucleotide (ODN), is designed to hybridise with a target sequence. The ORN can block chain elongation by a DNA polymerase and suppress amplification across the target sequence in a sequence-specific manner during PCR (ORNi-PCR). (**B**) ORNi-PCR can be applied to the detection of nucleotide differences. For example, if the target sequence includes a mutation (insertion, deletion or substitution), the ORN cannot efficiently hybridise with the mutated target sequence, resulting in amplification across the sequence. In this case, amplification indicates the presence of nucleotide differences in the target sequence.

We previously developed oligoribonucleotide (ORN) interference-PCR (ORNi-PCR) to inhibit amplification of a target DNA in a sequence-specific manner (Fig. 1A)^2^. In ORNi-PCR, an ORN (usually a 17–29 base RNA) inhibits amplification of a target DNA sequence containing a DNA sequence complementary to the ORN (Fig. 1A). DNA polymerases without 5′-3′ exonuclease activity (i.e., α-type) can be used for ORNi-PCR^2^. ORNs block elongation by DNA polymerases and are not used as primers themselves, although some DNA polymerases can potentially amplify DNA from RNA primers *in vitro*^3^. ORNi-PCR can be applied to detect nucleotide differences, such as insertion/deletion (indel) mutations introduced during genome editing, mutations causing diseases and single-nucleotide polymorphisms (SNPs; Fig. 1B)^4^. Because chemical synthesis of ORNs is inexpensive, it is affordable to screen a large number of cells possessing nucleotide differences using this method. ORNi-PCR can be performed on end-point levels as well as real-time levels using appropriate PCR machines^4^. Thus, ORNi-PCR is a useful method, but technical improvement could allow more precise discrimination of nucleotide differences in an ORN-specific manner for applications in molecular biology and medical fields^4^. In this regard, trial and error steps would still be necessary to find an optimal experimental condition for an ORN.

In the present study, we optimised the ORNi-PCR method to more precisely discriminate nucleotide differences using end-point levels. The optimised ORNi-PCR method, using two-step cycles (denaturation and annealing plus elongation), can discriminate single-nucleotide differences in genomic DNA (gDNA) and cDNA in an ORN-specific manner. In addition, we established a technical protocol for designing ORNi-PCR experiments, including establishing appropriate experimental conditions, and exploring different applications. The findings demonstrate that the developed Two-Step ORNi-PCR method is useful for simple, precise and cost-effective detection of nucleotide differences in various fields.

## Results and Discussion

### Comparison of Two- and Three-Step ORNi-PCR

**Figure 2:**
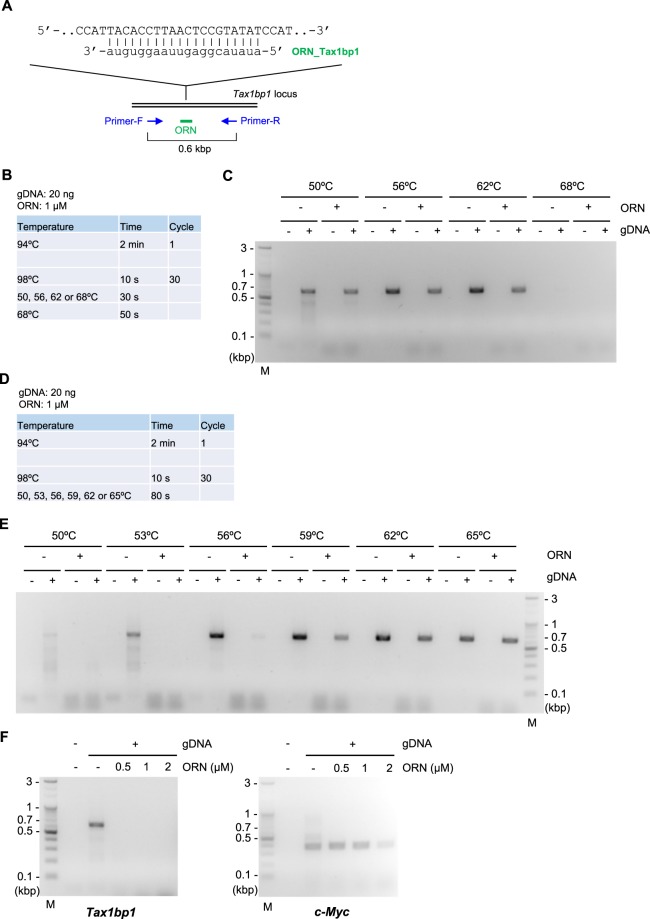
Suppression of target amplification by Two-Step ORNi-PCR. (**A**) Target position and nucleotide sequence of ORN_Tax1bp1, a 20 base ORN targeting the mouse *Tax1bp1* locus. The forward DNA sequence of the allele is shown. (**B**) Conditions for standard Three-Step ORNi-PCR. (**C**) Results of Three-Step ORNi-PCR. (**D**) Conditions for Two-Step ORNi-PCR. (**E**) Results of Two-Step ORNi-PCR. (**F**) Dose responses of ORN_Tax1bp1. ORNi-PCR was performed at an annealing plus elongation step temperature of 55 °C in the presence or absence of various concentrations of ORN_Tax1bp1. To investigate the specificity of the ORN, amplification of the c-*Myc* locus was also examined using genomic DNA (gDNA) extracted from Ba/F3 cells. M, molecular weight markers.

As reported previously^4^, ORNi-PCR can be used for detection of nucleotide differences (Fig. 1B). We therefore tested several ORNs and found that ORN_Tax1bp1, a 20 base ORN targeting the mouse *Tax1bp1* locus, did not suppress amplification across the target site at any annealing temperature, even below the predicted melting temperature (Tm) of the ORN, using ORNi-PCR with standard three-step cycles (Three-Step ORNi-PCR; Fig. 2A–C and Supplementary Tables S1 and S2). It is possible that ORN_Tax1bp1 cannot hybridise with the target site due to self-annealing during the annealing step. Alternatively, although the ORN likely hybridises with the target DNA sequence during the annealing step, it could become detached from the target at 68 °C during the elongation step, which is higher than the predicted Tm (Supplementary Table S2). To examine the latter possibility, we performed the Two-Step ORNi-PCR method, in which annealing and elongation are performed together in a single step (Fig. 2D). We found that ORN_Tax1bp1 effectively suppressed amplification across the target site when the annealing plus elongation step was performed at 50–56 °C (Fig. 2D and E). In addition, ORN_Tax1bp1 did not affect amplification of an irrelevant locus (c-*Myc*) at 0.5–2 μM although the presence of 2 μM of ORN_Tax1bp1 resulted in some non-specific suppression (Fig. 2F), confirming the target specificity of Two-Step ORNi-PCR. Thus, these results suggest that the hybridised ORN_Tax1bp1 does indeed detach from the target DNA at 68 °C during elongation in Three-Step ORNi-PCR, but effectively blocks elongation by DNA polymerase at a temperature below 56 °C in Two-Step ORNi-PCR.

**Figure 3:**
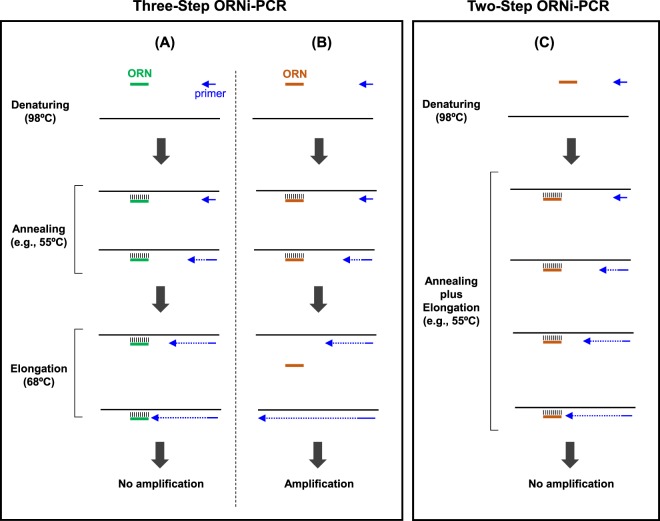
Schematic of Three-Step and Two-Step ORNi-PCR. (**A**) If the practical Tm of an ORN is higher than the temperature of the elongation step, the ORN can stably hybridise with the target DNA sequence, even during the elongation step, in a standard Three-Step ORNi-PCR, resulting in failure of target amplification. (**B**) If the practical Tm of an ORN is lower than the temperature of the elongation step, the hybridised ORN can detach from the target DNA sequence during the elongation step, resulting in successful target amplification. (**C**) In Two-Step ORNi-PCR, the annealing and elongation steps are performed at the same temperature, which is lower than the practical Tm of the ORN. Thus, the ORN stably hybridises the target DNA sequence during the annealing plus elongation step, resulting in failure of target amplification.

Based on the results shown in Fig. 2, another mode of ORNi-PCR could be possible (Fig. 3). If the practical Tm of an ORN is higher than 68 °C, the ORN could stably hybridise with the target site even at 68 °C during elongation in Three-Step ORNi-PCR (Fig. 3A). However, if the practical Tm of an ORN is lower than 68 °C, the hybridised ORN may detach from the target site during the elongation step, resulting in failure to suppress target amplification (Fig. 3B). Finally, in Two-Step ORNi-PCR, even if the practical Tm of an ORN is lower than 68 °C, the ORN could stably hybridise with the target site during the annealing plus elongation step performed at temperatures lower than the Tm, resulting in effective suppression of target amplification. Indeed, it was previously suggested that blocking PCR follows the same mode^5^.

### Calculation of the Tm of ORNs for Two-Step ORNi-PCR

To perform Two-Step ORNi-PCR with an optimal temperature for the annealing plus elongation step, it is necessary to calculate the Tm of ORNs. We calculated the Tm of ORNs using a previously reported formula^6–8^. As shown in Supplementary Table S2, the Tm of ORN_Tax1bp1 calculated using formula (I) [(a + u) * 2 + (g + c) * 4, where a, u, g and c are the number of A, U, G and C bases, respectively], and formula (II) [64.9 + 41 * (g + c − 16.4)/(a + u + g + c)], was 54 °C and 46 °C, respectively. Based on the results shown in Fig. 2, the practical Tm of ORN_Tax1bp1 was 53–56 °C, which is consistent with the Tm calculated by formula (I). Therefore, formula (I) appears to be more suitable than formula (II) for prediction of the practical Tm of ORNs.

To verify this result, we designed ORN_Tax1bp1_24b, a longer ORN targeting the mouse *Tax1bp1* locus (Supplementary Figure S1A). The Tm of ORN_Tax1bp1_24b calculated by formulae (I) and (II) was 66 °C and 52 °C, respectively (Supplementary Table S2). ORN_Tax1bp1_24b was not functional for Three-Step ORNi-PCR using an annealing temperature of 59–65 °C, as was the case for ORN_Tax1bp1 (Supplementary Figure S1B and C). By contrast, the ORN effectively suppressed PCR amplification in Two-Step ORNi-PCR using a temperature between 59 °C and 62 °C for the annealing plus elongation step (Supplementary Figure S1D and E). Therefore, the practical Tm of ORN_Tax1bp1_24b was 62–65 °C, which is close to the Tm of 66 °C calculated using formula (I). Thus, these results further indicate that formula (I) is suitable for prediction of the practical Tm of ORNs (Supplementary Figure S1F), and this ease of prediction facilitates the use of ORNi-PCR.

### Examination of optimal experimental conditions for Two-Step ORNi-PCR

**Figure 4:**
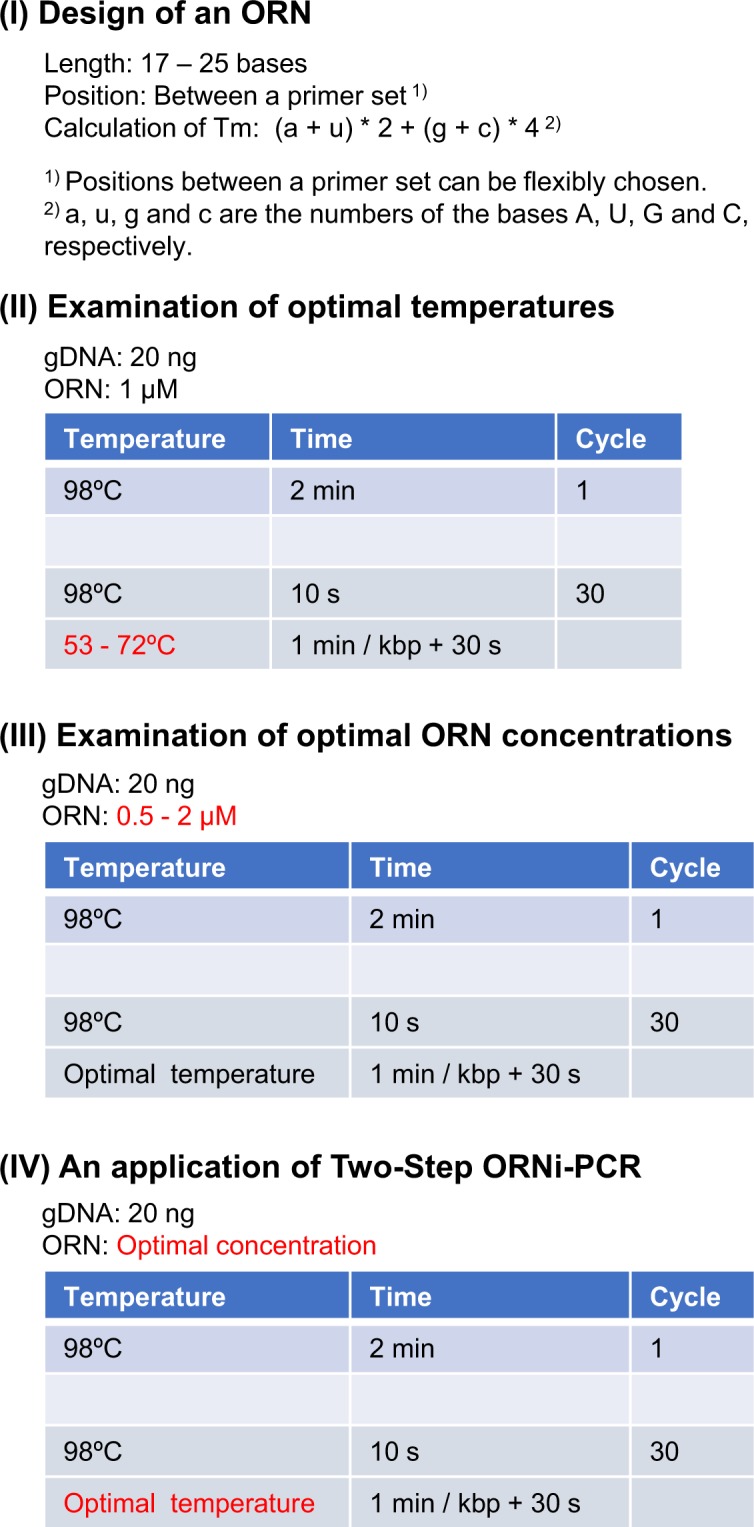
Step-by-step optimisation of Two-Step ORNi-PCR using KOD DNA polymerase. (**Step I**) Design of an ORN. (**Step II**) Determination of the optimal temperature. In this step, 1 μM ORN is used as a default. (**Step III**) Determination of the optimal ORN concentration. (**Step IV**) Practical application of Two-Step ORNi-PCR, such as detection of nucleotide mutations. Herein, we show an example using gDNA extracted from mammalian cells, for which 20 ng of gDNA and 30 cycles of denaturing and annealing plus elongation were used as a default. Other types of DNA (e.g., plasmids, fragments, cDNA) and α-type DNA polymerases (e.g., *Pfu* DNA polymerase) can be used for ORNi-PCR. Dependent on materials, the amount of DNA and the number of cycles of denaturing and annealing plus elongation may require modification.

Based on the results shown in Fig. 2 and Supplementary Figure S1, we suggest following step-by-step procedures for determination of optimal experimental conditions for Two-Step ORNi-PCR (Fig. 4). Based on our experience, the amount of gDNA (e.g., mammalian gDNA) and number of cycles in the annealing plus elongation step can be fixed as 20 ng and 30 cycles, respectively, as default values for ORNi-PCR using KOD DNA polymerase. These default values are basically preferable for detection of amplicons by electrophoresis. In the first step (Step I), an ORN of 17–25 bases is designed, and the Tm is calculated using formula (I). The calculated Tm should be 53–72 °C since the target sequence may not be efficiently amplified outside this temperature range (Fig. 2E). In the second step (Step II), the optimal temperature for the annealing plus elongation step is determined. In Two-Step ORNi-PCR, the annealing plus elongation step is performed at a temperature close to the predicted Tm (e.g., Tm ± 6 °C), and the Tm of primers is higher than the predicted Tm. In the third step (Step III), the optimal ORN concentration is determined. Two-Step ORNi-PCR is performed at an ORN concentration of 0.5–2 μM, since this is known to be an effective concentration range^4^. In the fourth step (Step IV), Two-Step ORNi-PCR is performed using the established optimal conditions for a given application, such as detection of nucleotide differences (Fig. 1B). In this regard, the positions of nucleotide mismatches in the hybridised DNA/ORN are not limited to the centre of the ORN^4^. The established protocol would be straightforward but may be time-consuming to find the most optimized condition. Although Tm of ORNs can be easily predicted, we found a few exceptions (Supplementary Table S2). In such cases, trial and error steps may be necessary to find an optimal Tm. Further optimisation of experimental conditions might be required dependent on materials (e.g., DNA, ORN, enzymes).

**Figure 5:**
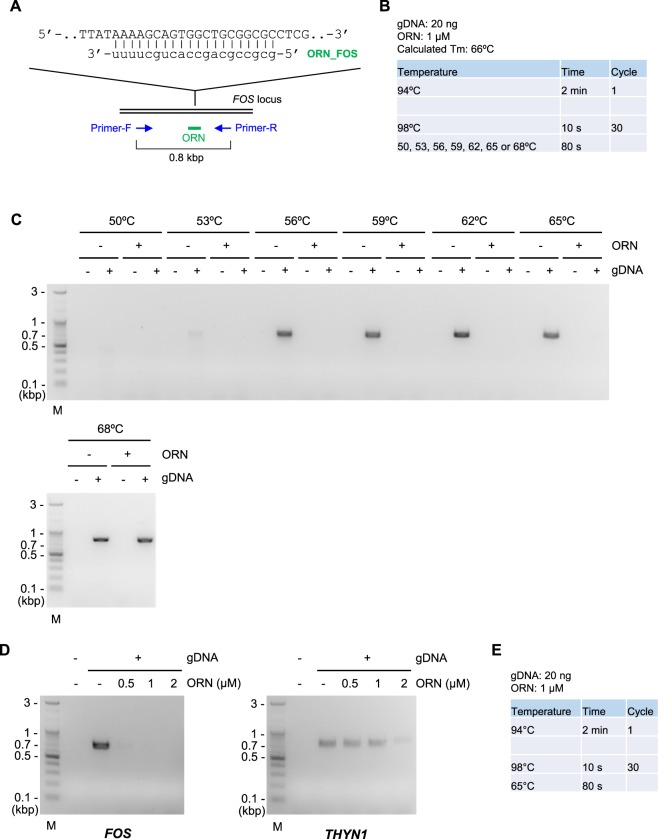
Confirmation of step-by-step optimisation of Two-Step ORNi-PCR. (**A**) Target position and nucleotide sequence of ORN_FOS, a 20 base ORN targeting the human *FOS* locus. The forward DNA sequence of the allele is shown. (**B**) Conditions for Two-Step ORNi-PCR. (**C**) Results of Two-Step ORNi-PCR. (**D**) Dose responses of ORN_FOS. ORNi-PCR was performed at an annealing plus elongation step temperature of 65 °C in the presence or absence of various concentrations of ORN_FOS. To investigate the specificity of the ORN, amplification of the *THYN1* locus was also examined. (**E**) Established conditions for Two-Step ORNi-PCR using ORN_FOS using gDNA extracted from 293T cells. M, molecular weight markers.

We examined the utility of the protocol outlined in Fig. 4. Following the protocol, we first designed ORN_FOS targeting the human *FOS* locus (Fig. 5A). Consistent with the calculated Tm of ORN_FOS (66 °C, Fig. 5B), the ORN was neither functional in Three-Step ORNi-PCR (Supplementary Figure S2) nor in Two-Step ORNi-PCR at an annealing plus elongation step temperature of 68 °C (Fig. 5C). By contrast, the ORN effectively suppressed amplification of the target locus in Two-Step ORNi-PCR at an annealing plus elongation step temperature of 56–65 °C (Fig. 5B and C). The practical Tm of the ORN was 65–68 °C, which is consistent with the calculated Tm. The ORN was effective and specific for Two-Step ORNi-PCR at a concentration of 0.5 or 1 μM (Fig. 5D). Thus, these results demonstrate that the protocol outlined in Fig. 4 is useful for Two-Step ORNi-PCR. The established optimal conditions for Two-Step ORNi-PCR with ORN_FOS are summarised in Fig. 5E.

In our previous study^4^, we unconsciously utilized Two-Step ORNi-PCR for detection of a single nucleotide insertion in the *CDKN2A(p16)* locus in HCT116. However, because we were unaware of importance of Tm of ORNs and the two-step setting, these parameters were not examined in detail. In the present study, we noticed such basic but important parameters through experiments and examined them in detail. We believe that the technical protocol established in the present study would be useful for researchers who use ORNi-PCR.

### Screening of genome-edited cells by Two-Step ORNi-PCR

**Figure 6:**
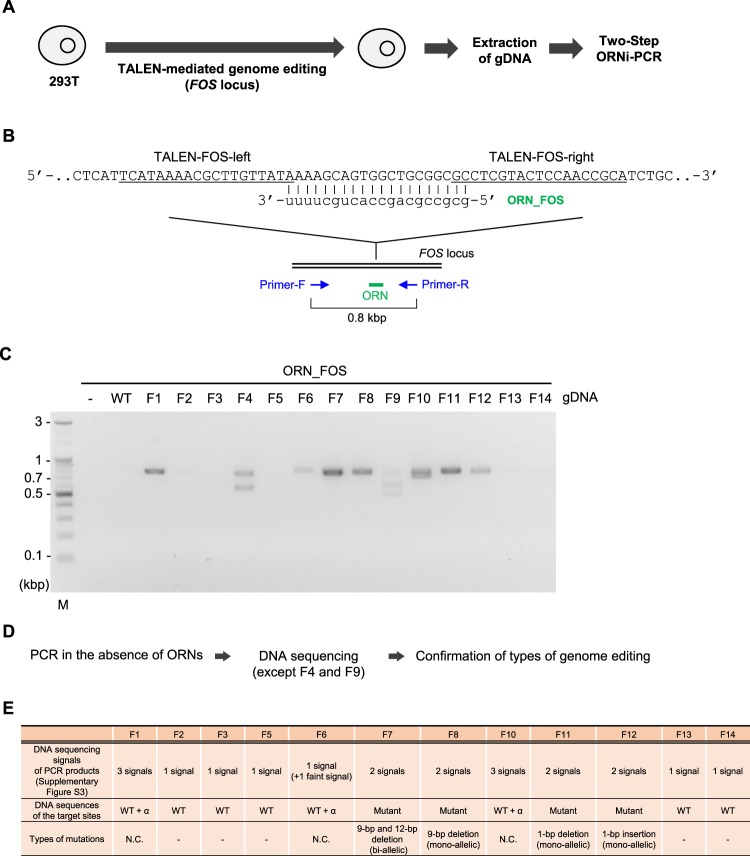
Screening of genome-edited cells by Two-Step ORNi-PCR. (**A**) Schematic diagram of the screening of genome-edited cells by Two-Step ORNi-PCR. (**B**) Target position of genome editing and sequence of ORN_FOS. The forward DNA sequence of the allele is shown. The target sites of TALEN-FOS-left and -right are underlined. Nucleotides in the spacer region surrounded by TALEN-FOS-left and -right are cleaved. (**C**) Results of Two-Step ORNi-PCR. The experimental conditions shown in Fig. 5E were used for Two-Step ORNi-PCR. M, molecular weight markers. (**D**) Schematic diagram of the confirmation of indel mutations. (**E**) Types of indel mutation. N.C., not confirmed.

We next applied Two-Step ORNi-PCR to discrimination of nucleotide differences introduced by genome editing. To this end, we established genome-edited 293 T cells in which the *FOS* locus was mutated in one or both alleles (Fig. 6A). Since the clustered regularly interspaced short palindromic repeats (CRISPR) system is the dominant tool for genome editing^9–11^, we previously used CRISPR for genome editing and showed that ORNi-PCR can be used to detect the introduced nucleotide mutations^4^. However, whether ORNi-PCR is able to detect indel mutations introduced by zinc finger nuclease (ZFN)^9^ or transcription activator-like effector nuclease (TALEN)^9^ remains unclear. We therefore employed TALEN-mediated genome editing to introduce indel mutations into the DNA sequence complementary with ORN_FOS in the *FOS* locus (Fig. 6B). We performed Two-Step ORNi-PCR using the established experimental conditions (Fig. 5E) to detect changes following genome editing. If the genome has been successfully edited at the target site, nucleotide mismatches would occur between the edited DNA sequence and ORN_FOS, resulting in failure of gDNA/ORN hybridisation at 65 °C during the annealing plus elongation step, and consequent amplification of the *FOS* locus. As shown in Fig. 6C, Two-Step ORNi-PCR successfully amplified products around the predicted position (0.8 kb) from gDNA in 9 out of 14 clones. More than two ORNi-PCR products were detected with clones F4 and F9, implying distinct bi-allelic or multiple-allelic genome editing in these clones. Meanwhile, clones F1, F6–F8 and F10–F12 yielded 0.8 kb products (Fig. 6C), indicating successful genome editing in these clones.

One potential limitation of ORNi-PCR is that it may be difficult to distinguish between mono-allelic and bi-allelic mutations using end-point levels^4^. Therefore, to characterise the types of mutations, we amplified the *FOS* locus from gDNA using these clones in the absence of ORNs, and directly sequenced the resulting amplicons (Fig. 6D and E). As shown in Supplementary Figures S3 and S4, clones F8, F11 and F12 harboured mono-allelic mutations, while F7 carried bi-allelic mutations. More than three different PCR products were detected using clones F1 and F10, indicating step-by-step genome editing, a mixture of different types of genome-edited cells or aneuploidy in cell cultures^4^. Although DNA sequencing of F6 also yielded two product signals, one was faint, possibly reflecting the presence of a small population of genome-edited cells in the sample (Fig. 6C). By contrast, only DNA sequencing signals corresponding to the intact TALEN target site were detected in amplicons from clones F2, F3, F5, F13 and F14 (Supplementary Figure S3), consistent with the results shown in Fig. 6C. Notably, faint bands were detected for clones F2, F13 and F14, and we cannot exclude the possibility that these samples might include a small population of genome-edited cells.

It is important to investigate whether Two-Step ORNi-PCR successfully amplified the edited but not the intact nucleotide sequences. To this end, we purified products from Two-Step ORNi-PCR amplification of clones F11 and F12 (mono-allelic single-nucleotide mutations; Supplementary Figure S4) from the agarose gel of electrophoresis (Fig. 6C). DNA sequencing of these products revealed signals only from edited nucleotide sequences (Supplementary Figure S5), confirming that Two-Step ORNi-PCR amplified edited but not intact nucleotide sequences. In addition, Two-Step ORNi-PCR could precisely discriminate single-nucleotide differences. Thus, Two-Step ORNi-PCR can be applied to screening of genome-edited cells.

To examine the importance of selection of the optimal temperature, we tested Two-Step ORNi-PCR using a lower temperature (60 °C) for the annealing plus elongation step (Supplementary Figure S6). Interestingly, Two-Step ORNi-PCR using gDNA from clone F11 (mono-allelic 1-base ‘deletion’) resulted in an amplicon, whereas amplification using clone F12 (mono-allelic 1-base ‘insertion’) did not yield a product (Supplementary Figure S6A–C). An RNA bulge may be formed in the ORN during hybridisation of gDNA(F11)/ORN_FOS (Supplementary Figure S6D), but failure to form a bulge may result in failure of hybridisation at 60 °C. By contrast, a DNA bulge may be formed more easily during hybridisation of gDNA(F12)/ORN_FOS (Supplementary Figure S6D), resulting in clear hybridisation at 60 °C. It is possible that different types of bulges may reflect the ease of hybridisation of ORNs with mutated target sequences, although further experiments are needed before such a conclusion can be drawn.

To detect successful genome editing, various end-point PCR-based methods have been developed^12,13^. These methods use a primer that is complementary to an intact target site to assess the presence of indel mutations, and inhibition of PCR amplification indicates introduction of indel mutations into the target site (negative detection methods)^12,13^. These methods can distinguish between mono- and bi-allelic mutations, even using end-point levels^12,13^, which may be an advantage over ORNi-PCR. However, when using such methods, an additional PCR amplification across the target site may be performed to confirm nucleotide sequences of indel mutations by DNA sequencing analysis. By contrast, ORNi-PCR does not require this additional PCR step because amplicons from ORNi-PCR can be directly sequenced. Like blocking PCR, ORNi-PCR can ignore superfluous DNA sequencing data and concentrate instead on useful information.

The CRISPR system of *Streptococcus pyogenes* has been most widely applied to genome editing. The cleavage site is between the third and fourth position from the protospacer adjacent motif (PAM)^14^. Although cleavage sites of TALEN and ZFN have also been defined, these are more variable^15^. We showed that ORNi-PCR can be applied for screening cells following genome editing by TALEN (Fig. 6), suggesting that ORNi-PCR can detect genome editing even if cleavage sites are not strictly defined. In this regard, an ORN should be designed so that its centre is located close to a cleavage site hot spot.

### Detection of point mutations by Two-Step ORNi-PCR

**Figure 7:**
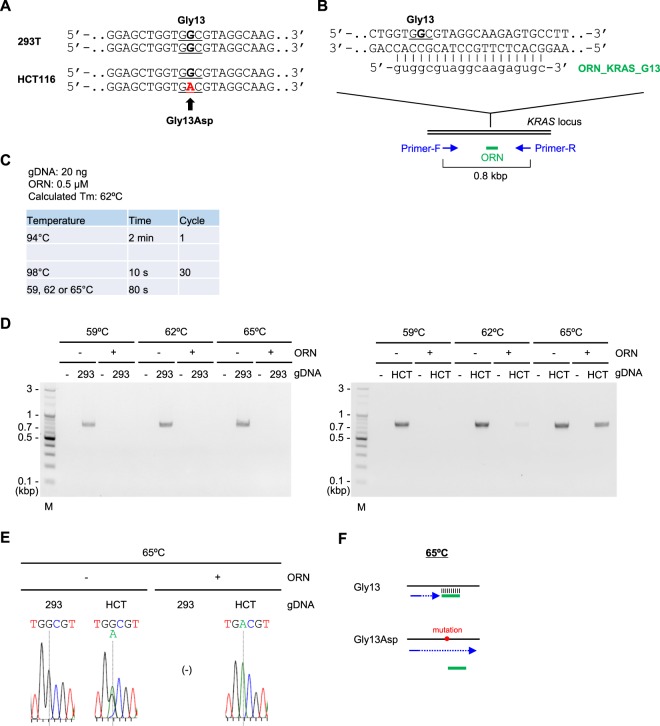
Discrimination of a single-nucleotide difference in the *KRAS* locus by Two-Step ORNi-PCR. (**A**) Nucleotide mutation leading to a Gly13Asp amino acid change in the human *KRAS* locus in HCT116 cells. Forward DNA sequences around the Gly13 position (underlined) of both alleles are shown. (**B**) Target position of ORN_KRAS_G13, a 19 base ORN targeting the human *KRAS* locus. Forward and reverse DNA sequences around the Gly13 position (underlined) of one allele are shown. (**C**) Conditions for Two-Step ORNi-PCR using ORN_KRAS_G13. (**D**) Results of Two-Step ORNi-PCR. M, molecular weight markers. (**E**) DNA sequencing of Two-Step ORNi-PCR products from (**D**) purified from agarose gels and subjected to DNA sequencing analysis using a forward primer. (**F**) Mode of discrimination of a single-nucleotide substitution by Two-Step ORNi-PCR. ORN_KRAS_G13 (green) hybridises with the intact target site, but not the target site including the Gly13Asp mutation, at an annealing plus elongation step temperature of 65 °C.

We next examined whether Two-Step ORNi-PCR could discriminate point mutations (substitutions) in cancer cells. To this end, we utilised the *KRAS* locus in human colon cancer cell line HCT116, in which a single nucleotide corresponding to glycine 13 (Gly13) is mutated from G to A in one allele (Fig. 7A). To detect the mutation by Two-Step ORNi-PCR, we designed ORN_KRAS_G13, an ORN complementary to the intact sequence but with a single-nucleotide mismatch with the corresponding DNA sequence in the other allele (Fig. 7B). The ORN can form a nucleotide mismatch with the complementary DNA sequence at the fourth position from its 5′ end. In this regard, we previously reported that it is not necessary to form a nucleotide mismatch around the centre of an ORN for ORNi-PCR^4^. As a positive control (to confirm suppression of target amplification by the ORN), we used gDNA from 293 T cells in which the corresponding site is intact in both alleles (Fig. 7A).

Based on the protocol outlined in Fig. 4, we tested ORN_KRAS_G13 at several different temperatures for the annealing plus elongation step (Fig. 7C). The results showed that Two-Step ORNi-PCR with 0.5 μM ORN_KRAS_G13 suppressed amplification of the intact *KRAS* locus from 293T gDNA at 59–65 °C but successfully amplified the mutated *KRAS* locus from HCT116 gDNA at 65 °C (Fig. 7D), implying sequence-specific suppression of target amplification. In fact, DNA sequencing analysis of a Two-Step ORNi-PCR product revealed signals corresponding to the mutated sequence but not the intact sequence (Fig. 7E). Thus, Two-Step ORNi-PCR can also be used to detect a point mutation (substitution). When the ORN_KRAS_G13 concentration was 1 μM, the mutated *KRAS* locus of HCT116 was also suppressed (Supplementary Figure S7). Notably, Two-Step ORNi-PCR with 0.5 μM ORN_KRAS_G13 completely suppressed amplification of the *KRAS* locus, even from HCT116 gDNA at an annealing plus elongation temperature of 59 °C (Fig. 7D). In addition, Two-Step ORNi-PCR did not suppress amplification from 293 T gDNA at 68 °C (Supplementary Figure S8A and B). These results also indicate the importance of selection of an optimal temperature for the annealing plus elongation step to precisely discriminate single-nucleotide differences (Fig. 7F and Supplementary Figure S8C).

**Figure 8:**
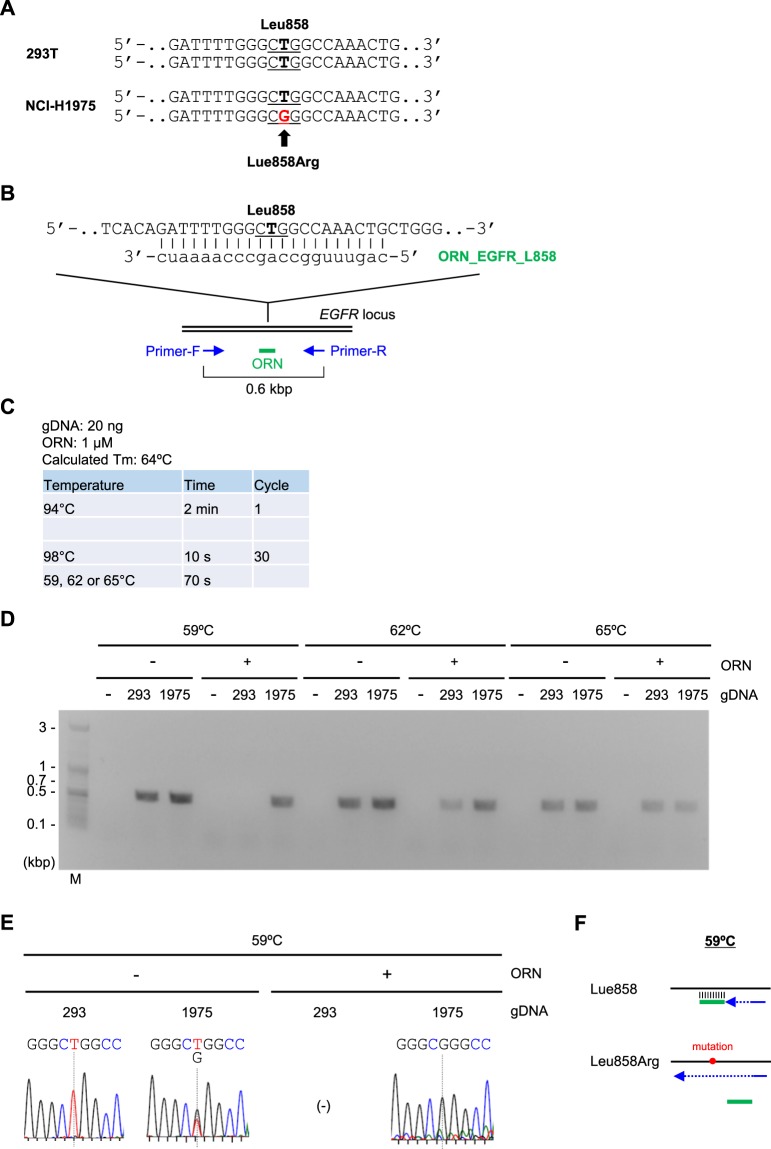
Discrimination of a single-nucleotide difference in the *EGFR* locus by Two-Step ORNi-PCR. (**A**) Nucleotide mutation leading to a Leu858Arg amino acid change in the human *EGFR* locus in NCI-H1975 cells. Forward DNA sequences around the Leu858 position (underlined) of both alleles are shown. (**B**) Target position of ORN_EGFR_L858, a 21 base ORN targeting the human *EGFR* locus. The forward DNA sequence around the Leu858 position (underlined) of one allele is shown. (**C**) Conditions for Two-Step ORNi-PCR using ORN_EGFR_L858. (**D**) Results of Two-Step ORNi-PCR. M, molecular weight markers. (**E**) DNA sequencing of Two-Step ORNi-PCR products from (**D**) purified from agarose gels and subjected to DNA sequencing analysis using a forward primer. (**F**) Mode of discrimination of a single-nucleotide difference by Two-Step ORNi-PCR. ORN_EGFR_L858 hybridises with the intact target site but not the mutated site at an annealing plus elongation step temperature of 59 °C.

As another example, we tested Two-Step ORNi-PCR for discrimination of a single-nucleotide substitution at the position in the codon corresponding to leucine 858 (Leu858) in the *EGFR* locus in human lung cancer cell line NCI-H1975 (Fig. 8A and B). The results showed that Two-Step ORNi-PCR suppressed amplification of the intact but not the mutated *EGFR* locus sequence from NCI-H1975 gDNA in the presence of 1 μM of ORN_EGFR_L858 at 59 °C (Fig. 8C–F).

**Figure 9:**
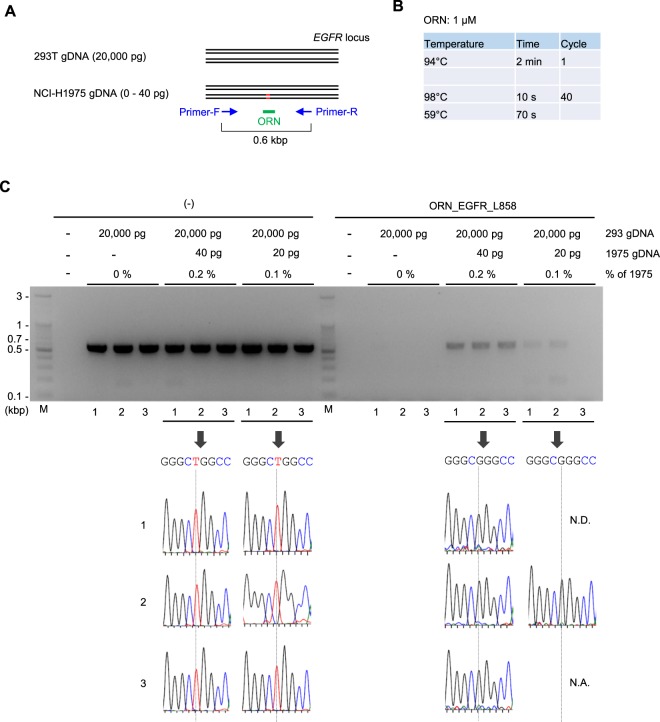
Detection of a minor population of cells harboring the mutated *EGFR* locus by Two-Step ORNi-PCR. (**A**) Schematic diagram of the detection of a minor population of cells harboring the mutated *EGFR* locus by Two-Step ORNi-PCR. (**B**) Conditions for Two-Step ORNi-PCR using ORN_EGFR_L858. (**C**) (Upper) Results of Two-Step ORNi-PCR. M, molecular weight markers. (Lower) DNA sequencing of Two-Step ORNi-PCR products purified from agarose gels and subjected to DNA sequencing analysis using a forward primer. N.D., not detected; N.A., not analysed.

Next, we examined sensitivity of Two-Step ORNi-PCR. For cancer diagnosis, it would be important to detect presence of a small population of cancer cells possessing causative nucleotide mutations among a major population of normal cells. As a model study, we mixed 293 T and NCI-H1975 gDNAs [20 ng (20,000 pg) and 0–40 pg, respectively] and analysed how sensitively ORNi-PCR could detect the single nucleotide mutation corresponding to Leu858Arg in the *EGFR* locus (Fig. 9A). In the present study, we performed ORNi-PCR with 40 cycles of denaturing and annealing plus elongation to detect the small population of the mutated *EGFR* locus (Fig. 9B). Standard PCR followed by DNA sequencing analysis failed to detect presence of a small population (0.2 or 0.1%) of NCI-H1975 gDNA (Fig. 9C). By remarkable contrast, Two-Step ORNi-PCR amplified only the mutated *EGFR* locus when 0.2% of NCI-H1975 gDNA was mixed (Fig. 9C). Two-Step ORNi-PCR amplified the *EGFR* locus even when 0.1% of NCI-H1975 gDNA was mixed, although one of three ORNi-PCR failed in amplification. DNA sequencing analysis for an amplicon showed signals corresponding to the mutated but not intact *EGFR* sequence. Notably, the intact *EGFR* locus was never visibly amplified even by 40 cycles of denaturing and annealing plus elongation in ORNi-PCR. Taken together, these results demonstrate that ORNi-PCR can accurately detect presence of at least 0.2% of the cancer cell possessing a heterozygous single nucleotide mutation in a target locus.

We had been using ORNs purified by high-performance liquid chromatography (HPLC) for ORNi-PCR, and it would be more cost-effective if an HPLC purification step was not required. We therefore tested desalted rather than HPLC-purified ORN_KRAS_G13 in Two-Step ORNi-PCR under the experimental conditions that yielded the results shown in Fig. 7C (Supplementary Figure S9A). The results with desalted ORN_KRAS_G13 (Supplementary Figure S9B and C) were comparable to those obtained using HPLC-purified ORN_KRAS_G13 (Fig. 7D and E). Thus, desalted ORNs can be used for ORNi-PCR, lowering the experimental cost. In this context, although blocking PCR using artificial nucleic acids such as LNAs and PNAs can be used for the same purpose^16,17^, preparation of artificial nucleic acids is more expensive. Synthesis of LNAs and PNAs cost at least more than 4- and 10-fold higher than that of RNAs, respectively. In addition, as to PNAs, limitation on purine/pyrimidine ratio and solubility is known^18^. DNA can be synthesised more affordably and used as blocking nucleotides in blocking PCR^1,5^. However, DNA would need to be modified at the 3′ end to avoid extension, adding cost, and high-fidelity DNA polymerases retaining 3′-5′ exonuclease activity cannot be used because they may remove such modifications.

### Application of Two-Step ORNi-PCR to the detection of nucleotide differences in cDNA

**Figure 10:**
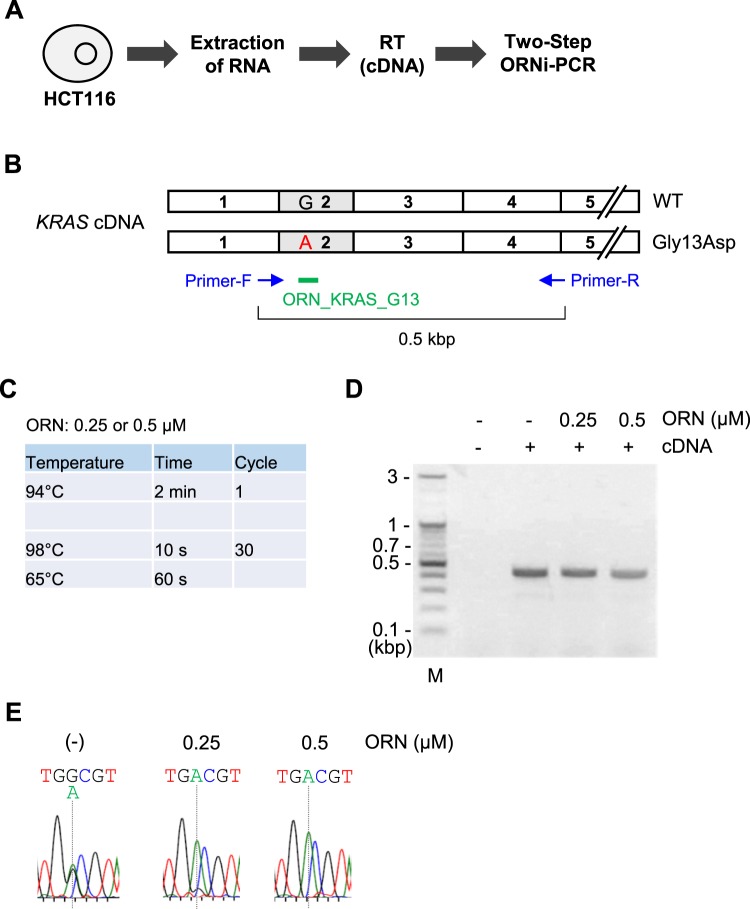
Two-Step ORNi-PCR using cDNA to discriminate a single-nucleotide difference. (**A**) Schematic diagram of Two-Step ORNi-PCR with cDNA as template. After extraction of total RNA from HCT116 cells, RNA was reverse-transcribed and the resulting cDNA was used as template for Two-Step ORNi-PCR. (**B**) Schematic diagram of human *KRAS* cDNA prepared from HCT116 cells. (**C**) Conditions for Two-Step ORNi-PCR using cDNA. (**D**) Results of Two-Step ORNi-PCR. M, molecular weight markers. (**E**) DNA sequencing of Two-Step ORNi-PCR products from (**D**) purified from agarose gels and subjected to DNA sequencing analysis using a forward primer.

ORNi-PCR may be used with DNAs other than gDNA, such as cDNA. We therefore examined whether ORNi-PCR can be used for sequence-specific suppression of target amplification from cDNA. To this end, we extracted total RNA from HCT116 cells, performed reverse transcription (RT) (Fig. 10A) and attempted to detect a single-nucleotide mutation in the resulting *KRAS* cDNA by Two-Step ORNi-PCR with ORN_KRAS_G13 (Fig. 10B). A primer set across exons was used to avoid amplification of the *KRAS* locus from gDNA (Fig. 10B). Based on the results in Fig. 7, the annealing plus elongation step was performed at 65 °C (Fig. 10C and D), and amplicons were subjected to DNA sequencing analysis. As shown in Fig. 10E, sequencing signals derived from both the intact and Gys13Asp-mutated sequences were detected in the absence of ORN_KRAS_G13, suggesting that the *KRAS* gene was transcribed from both alleles in HCT116 cells. When 0.25 or 0.5 μM ORN_KRAS_G13 was used for Two-Step ORNi-PCR, the sequencing signal derived from the intact sequence was dramatically decreased in a dose-dependent manner (Fig. 10E). These results clearly indicate that ORNi-PCR can be applied to suppression of target amplification from cDNA in an ORN-specific manner. We also tested lower (59 °C) and higher (68 °C) temperatures for the annealing plus elongation step (Supplementary Figure S10). The lower temperature suppressed amplification from both alleles, while the higher temperature did not effectively suppress target amplification, consistent with the results of Two-Step ORNi-PCR using gDNA (Fig. 7 and Supplementary Figure S8).

**Figure 11:**
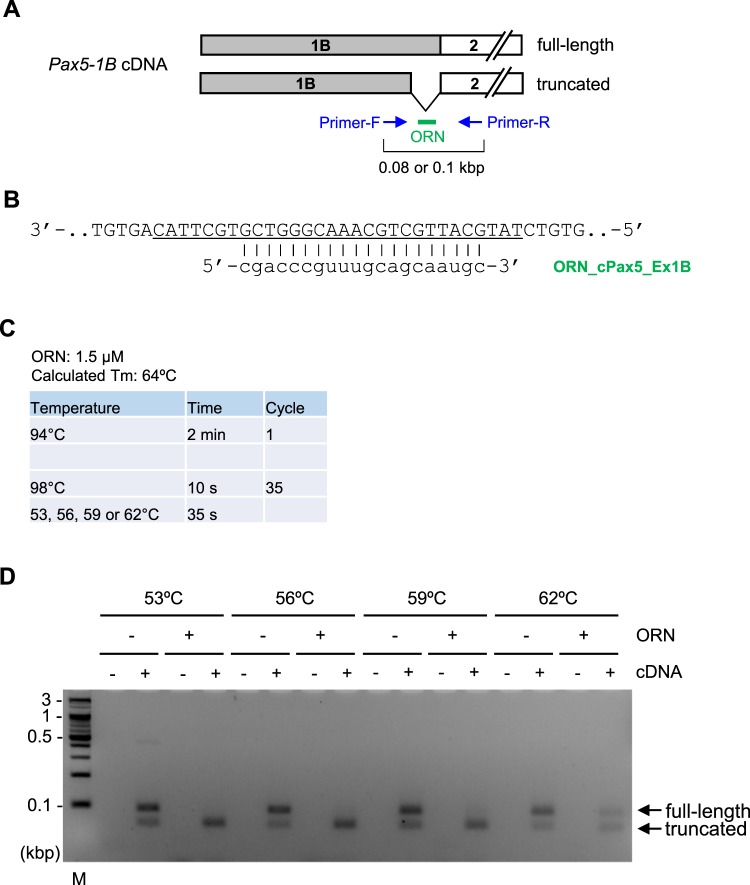
Two-Step ORNi-PCR using cDNA to discriminate splice variants. (**A**) Schematic diagram of *Pax5-1B* cDNA prepared from DT40 cells. Two forms of transcripts including exon 1B are produced from the chicken *Pax5* gene in DT40 cells. A part of the exon 1B region is truncated in the shorter form (truncated). (**B**) Target position of ORN_cPax5_Ex1B, a 20 base ORN targeting exon 1B of the chicken *Pax5* locus. The cDNA sequence of *Pax5-1B* (full-length) is shown. The underlined sequence is truncated in *Pax5-1B* (truncated) cDNA. (**C**) Conditions for Two-Step ORNi-PCR using cDNA. (**D**) Results of Two-Step ORNi-PCR. The predicted positions of each amplicon are indicated by arrows (full-length and truncated forms). M, molecular weight markers.

We also focused on a splice variant of the chicken *Pax5* gene, *Pax5-1B*, in which exon 1B is the first exon^19^. In our previous study^19^, we found that two forms (full-length or truncated) of *Pax5-1B* transcript were produced from the gene in the chicken B cell line DT40 (Fig. 11A). In the present study, we attempted to suppress amplification from the full-length but not the truncated form of *Pax5-1B* cDNA by ORNi-PCR. We designed ORN_cPax5_Ex1B to be complementary to the full-length but not the truncated form (Fig. 11A and B) and carried out Two-Step ORNi-PCR following the protocol in Fig. 4. As shown in Supplementary Figure S11, Two-Step ORNi-PCR with 1 μM ORN_cPax5_Ex1B resulted in partial suppression of amplification from the *Pax5-1B* (full-length) cDNA at an annealing plus elongation step temperature of 53–59 °C. However, when 1.5 μM ORN was used, Two-Step ORNi-PCR effectively suppressed amplification from the *Pax5-1B* (full-length) but not the truncated form at an annealing plus elongation step temperature of 53–59 °C (Fig. 11C and D). Thus, ORNi-PCR can be applied to target amplification of a desirable transcriptional variant.

The amount of target transcription is dependent on several factors such as the transcription rate in cells. Therefore, the amount of target cDNA in ORNi-PCR can differ markedly from that of gDNA, and further optimisation of ORN concentration and the number of PCR cycles may be required. However, as shown in Figs 10 and 11, the established protocol for Two-Step ORNi-PCR with gDNA can be applied when using cDNA. Regarding the copy number of a target sequence, even for a single-copy locus, a number of target cDNAs may be acquired from a single cell. Therefore, ORNi-PCR using cDNA may be advantageous when disposable materials (e.g., cells) are limited. If a target locus containing nucleotide mutations is not transcribed, such mutations may not be physiologically important. In this regard, it is more physiologically significant to analyse nucleotide mutations in transcripts rather than gDNA. It was reported that long RNAs (750 bases) can be used to suppress target amplification in PCR and applied to amplification of a splice variant of interest^20^. This method is useful, but it would be cumbersome to acquire such long RNAs via *in vitro* transcription. Meanwhile, ORNi-PCR can be used for the same purpose without such cumbersome procedures, representing an advantage over the long RNA-based method.

## Conclusions

In this study, we refined ORNi-PCR and established the Two-Step ORNi-PCR method for precise discrimination of nucleotide differences (Figs 2–5). In addition, we established a step-by-step experimental protocol for optimisation of Two-Step ORNi-PCR (Fig. 4). The Tm of ORNs can be easily predicted, and the predicted Tm is generally close to the practical Tm, with a few exceptions (Supplementary Table S2). Following the established protocol, we succeeded in screening genome-edited cells (Fig. 6) and discriminating nucleotide mutations in cancer cells at the single-nucleotide level (Figs 7–9). In addition, we demonstrated that Two-Step ORNi-PCR can be used with both gDNA and cDNA templates (Figs 10 and 11), consistent with its use in a broad range of applications in molecular biology and medicine. In our previous study^4^, we showed that ORNi-PCR (Three-Step ORNi-PCR) is a useful method for discrimination of nucleotide differences, but time-consuming trial and error steps would still be necessary to find an optimal experimental condition for an ORN. However, in the present study, we demonstrated that Two-Step ORNi-PCR is more straightforward and practical than Three-Step ORNi-PCR. ORNs can be designed flexibly^2^ and synthesised in a cost-effective manner. In addition to end-point ORNi-PCR, real-time ORNi-PCR is feasible^2,4^. We believe that Two-Step ORNi-PCR is a useful method for simple, precise, cost-effective and positive detection of nucleotide differences.

## Methods

### Oligonucleotides

Primers used in this study are listed in Supplementary Table S1. ORNs were chemically synthesised, and all ORNs other than those shown in Supplementary Figure S9 were purified by HPLC (FASMAC). ORNs are listed in Supplementary Table S2.

### ORNi-PCR with gDNA

gDNA was extracted from Ba/F3, 293 T, HCT116, and NCI-H1975 cells by standard phenol/chloroform extraction. ORNi-PCR was performed with KOD-Plus-Ver. 2 (Toyobo) in mixtures containing 20 pg–20 ng of gDNA, 0.3 µM of each primer and 0.5–2 µM ORN prepared in a 10 μl volume according to the manufacturer’s protocol using Mastercycler pro S (Eppendorf). For ORNi-PCR using standard three-step cycles, reactions were carried out with an initial denaturation at 94 °C for 2 min, followed by 30 cycles at 98 °C for 10 s, 53–68 °C for 30 s and 68 °C for 50 s. For Two-Step ORNi-PCR, reactions were carried out with an initial denaturation at 94 °C for 2 min, followed by 30–40 cycles at 98 °C for 10 s and 53–68 °C for 70–80 s. ORNi-PCR products were electrophoresed on 1% or 2% agarose gels and, where necessary, subjected to DNA sequencing. DNA sequencing data were analysed using Applied Biosystems Sequence Scanner Software v2.0 (Thermo Fisher Scientific).

### Genome editing

293T cells were cultured in DMEM medium (Wako) supplemented with 10% foetal bovine serum. TALEN was used for genome editing of the human *FOS* locus. TALEN plasmids targeting the human *FOS* gene (TALEN-FOS-left and TALEN-FOS-right) were designed and purchased from Thermo Fisher Scientific. 293 T cells (4 × 10^5^) were transfected with TALEN expression plasmids (4 µg each) and 0.4 µg of pcDNA3.1/Hygro(−) (Thermo Fisher Scientific) using Lipofectamine 3000 (Thermo Fisher Scientific). After 2 days, hygromycin B was added (0.4 mg/ml) and hygromycin-resistant colonies were picked and cultured.

### RNA extraction, RT and Two-Step ORNi-PCR with cDNA

Total RNA was extracted from HCT116 or DT40 cells using Isogen II (Nippon Gene). RNA (125 ng) was used for RT with ReverTra Ace qPCR RT Master Mix (Toyobo) in a 10 μl volume. After the RT reaction, cDNA was diluted with 40 μl of distilled water and ORNi-PCR was performed using KOD-Plus-Ver. 2. ORNi-PCR mixtures containing 1 μl of cDNA, 0.3 µM of each primer and 0.25–1.5 µM ORN were prepared in a 10 μl volume according to the manufacturer’s protocol. Reactions were carried out with an initial denaturation at 94 °C for 2 min, followed by 30–35 cycles at 98 °C for 10 s and 53–68 °C for 35–60 s.

## Electronic supplementary material

Supplementary Information
