# Supplementary material for: A refined two-step oligoribonucleotide interference-PCR method for precise discrimination of nucleotide differences
Source: Sci Rep. 2018 Nov 21;8:17195. doi: 10.1038/s41598-018-35479-0 (PMC6249316; doi:10.1038/s41598-018-35479-0)
Supplement: Supplementary file 1 — Supplementary Information [file 41598_2018_35479_MOESM1_ESM.pdf]

Supplementary Text

A refined two-step oligoribonucleotide interference-PCR method for precise discrimination of nucleotide differences

Toshitsugu Fujita, Miyuki Yuno, Fusako Kitaura and Hodaka Fujii

Supplementary Table and Figure Legends

Supplementary Table S1. Primers used in this study

Supplementary Table S2. Information on ORNs

Supplementary Figure S1. Prediction of the Tm of ORNs.

(**A**) Target position and nucleotide sequence of ORN_Tax1bp1_24b, a 24 base ORN targeting the mouse *Tax1bp1* locus. The forward DNA sequence of the allele is shown. Nucleotides additional to ORN_Tax1bp1 are shown in red. (**B**) Conditions for standard Three-Step ORNi-PCR. (**C**) Results of Three-Step ORNi-PCR. (**D**) Conditions for Two-Step ORNi-PCR. (**E**) Results of Two-Step ORNi-PCR. (**F**) Predicted Tm of ORNs. (1) Tm calculated based on the following formula: $(a + u) * 2 + (g + c) * 4$. (2) Tm calculated based on the following formula: $64.9 + 41 * (g + c - 16.4) / (a + u + g + c)$. In both formulae, a, u, g and c are the number of the A, U, G and C bases, respectively. Experiments were performed using gDNA extracted from Ba/F3 cells. M, molecular weight markers.

Supplementary Figure S2. Amplification of the *FOS* locus is not suppressed in Three-Step ORNi-PCR.

(**A**) Conditions for Three-Step ORNi-PCR. (**B**) Results of Three-Step ORNi-PCR. gDNA extracted from 293T cells was used. M, molecular weight markers.

Supplementary Figure S3. PCR examination of indel mutations in the *FOS* locus.

(**A**) Results of PCR performed in the absence of ORN_FOS with gDNA extracted from wild-type (WT) and genome-edited 293T cells. (**B**) Nucleotide sequences around the TALEN_FOS target site. Forward and reverse DNA sequences of one allele are shown. TALEN_FOS-left and -right binding sites are underlined. Nucleotide sequences shown in (**C**) start from the dotted green arrow. (**C**) DNA sequencing of PCR products from the WT and clones F1–F14 (apart from F4 and F9) from (**A**) purified from agarose gels and subjected to DNA sequencing analysis using a reverse primer.

Supplementary Figure S4. DNA sequences around the TALEN_FOS target sites in WT and genome-edited 293T cells.

Forward DNA sequences of both alleles are shown, and TALEN_FOS-left and -right binding sites are underlined. The target site of ORN_FOS is highlighted in light green. See also Supplementary Figure S3.

Supplementary Figure S5. DNA sequencing signals from ORNi-PCR products.

ORNi-PCR products from clones F11 and F12 in Figure 6C were purified from agarose gels and subjected to DNA sequencing analysis using a reverse primer.

Supplementary Figure S6. Two-Step ORNi-PCR without temperature optimisation.

(**A**) Conditions for Two-Step ORNi-PCR for the *FOS* locus. A lower annealing plus elongation step temperature was tested for Two-Step ORNi-PCR. (**B**) Results of Two-Step ORNi-PCR. M, molecular weight markers. (**C**) Mode of ORN-mediated suppression of target amplification for clones F11 and F12. At the optimal temperature for the annealing plus elongation step (65°C), ORN_FOS does not hybridise with the mutated target sites in clones F11 and F12. At an annealing plus elongation step temperature of 60°C (5°C lower than the optimal temperature), ORN_FOS hybridises with the single base insertion target site in F12, but not with the single base deletion target site in F11. (**D**) Mode of single-nucleotide skipping for hybridisation of gDNA/ORN_FOS. RNA and DNA bulges can form during hybridisation.

Supplementary Figure S7. Two-Step ORNi-PCR for the *KRAS* locus.

(**A**) Conditions for Two-Step ORNi-PCR with 1 μM ORN_KRAS_G13. (**B**) Results of Two-Step ORNi-PCR. M, molecular weight markers.

Supplementary Figure S8. Two-Step ORNi-PCR for the *KRAS* locus.

(**A**) Conditions for Two-Step ORNi-PCR. A higher annealing plus elongation step temperature is used for Two-Step ORNi-PCR. (**B**) Results of Two-Step ORNi-PCR. M, molecular weight markers. (**C**) Mode of ORN-mediated suppression of target amplification for the *KRAS* locus. At an annealing plus elongation step temperature of 59°C (lower than the optimal temperature), ORN_KRAS_G13 hybridises with both the intact target site and the single base substitution target site. At the optimal temperature for the annealing

plus elongation step (65°C), ORN_ KRAS_G13 hybridises with the intact target site but not the single base substitution site. At an annealing plus elongation step temperature higher than the optimal temperature (68°C), ORN_KRAS_G13 hybridises with neither the intact target site nor the single base substitution site. The mode for 65°C is also shown in Figure 7F.

Supplementary Figure S9. Discrimination of a single-nucleotide difference by Two-Step ORNi-PCR with a desalted ORN.

(**A**) Conditions for Two-Step ORNi-PCR with ORN_KRAS_G13 purified by desalting rather than HPLC. (**B**) Results of Two-Step ORNi-PCR. M, molecular weight markers. (**C**) DNA sequencing of Two-Step ORNi-PCR products from (**B**) purified from agarose gels and subjected to DNA sequencing analysis using a forward primer.

Supplementary Figure S10. Two-Step ORNi-PCR using cDNA to discriminate a single-nucleotide difference in *KRAS*.

(**A**) Conditions of Two-Step ORNi-PCR using cDNA from HCT116 cells and ORN_KRAS_G13. (**B**) Results of Two-Step ORNi-PCR. M, molecular weight markers. (**C**) DNA sequencing of Two-Step ORNi-PCR products from (**B**) purified from agarose gels and subjected to DNA sequencing analysis using a forward primer.

Supplementary Figure S11. Two-Step ORNi-PCR using cDNA to discriminate splice variants.

(**A**) Conditions for Two-Step ORNi-PCR using cDNA from DT40 cells and ORN_cPax5_Ex1B. (**B**) Results of Two-Step ORNi-PCR. The predicted positions of each amplicon are indicated by arrows for full-length and truncated forms. M, molecular weight markers.

Supplementary Table S1

Number	Name	Sequence (5' → 3')	Experiments
28161	mTax1bp1-exon2-F2	ttgactgagttgtatccccatcc	Figure 2 and Supplementary Figure S1 (Tax1bp1)
28162	mTax1bp1-exon2-R2	tgcacagtgtttagtatttcatggtg	Figure 2 and Supplementary Figure S1 (Tax1bp1)
28218	mc-myc_-0.6k-F	ggtcgttctggaaagaatgtgc	Figure 2F (c-Myc)
28221	mc-myc_-0.4k-R	cttgccctgcgtatatcagtcac	Figure 2F (c-Myc)
27264	hc-fos-prom-F	aactgtcttcagtttccgtacaagg	Figures 5 and 6, and Supplementary Figures S2, S3 and S6 (FOS)
27265	hc-fos-prom-R	gggtgagtggtagtaagagaggcta	Figures 5 and 6, and Supplementary Figures S2, S3 and S6 (FOS)
28224	hTHYN1-gRNA-target-15-F5	ccgcagtcgagtctgcagagtgttgg	Figure 5D (THYN1)
28225	hTHYN1-gRNA-target-15-R5	caaggctgggctcaaattccacatcc	Figure 5D (THYN1)
28246	hKRAS-F3	tagaggtgggggtccactaggaaaact	Figure 7 and Supplementary Figures S7-9 (KRAS)
28247	hKRAS-R3	cacttccaatcaaaatgcacagagagtg	Figure 7 and Supplementary Figures S7-9 (KRAS)
28248	hKRAS-cDNA-F	cgggagagaggcctgctgaaaat	Figure 10 and Supplementary Figure S10 (KRAS)
28249	hKRAS-cDNA-R	ggcatcatcaacaccctgtcttgtc	Figure 10 and Supplementary Figure S10 (KRAS)
28250	hEGFR-Exon21-F	gcctttccattctttggatcag	Figures 8 and 9 (EGFR)
28251	hEGFR-Exon21-R	ctgcagggagagactgaaacct	Figures 8 and 9 (EGFR)
26540	cPax5-ex1B-F3	gccccgatggaaatacactg	Figure 11 and Supplementary Figure S11 (Pax5-1B)
26542	cPax5-ex2-R2	ggcggccattcacaaaaac	Figure 11 and Supplementary Figure S11 (Pax5-1B)

Supplementary Table S2

Name	Sequence (5' → 3')	Target locus	Length (bases)	Predicted Tm (ºC) (1)	Predicted Tm (ºC) (2)	Practical Tm (ºC)	68°C for the elongation step	References
ORN_Tax1bp1	auauacggaguuaaggugua	mouse *Tax1bp1*	20	54	46	53–56	Not acceptable	This study
ORN_Tax1bp1_24b	ggauauacggaguuaagguguaau	mouse *Tax1bp1*	24	66	52	62–65	Not acceptable	This study
ORN_FOS	gcgccgcagccacugcuuuu	human *FOS*	20	66	58	65–68	Not acceptable	This study
ORN_KRAS_G13	guggcguaggcaagagugc	human *KRAS*	19	62	55	62–68	Not acceptable	This study
ORN_EGFR_L858	caguuuggccagcccaaaauc	human *EGFR*	21	64	54	59–62	Not acceptable	This study
ORN_cPax5_Ex1B	cgacccguuugcagcaaugc	chicken *Pax5*	20	64	56	59–62	Not acceptable	This study
ORN_302F	ccgggggcgcugggcuguccc	human *IRF-1*	21	78	68	Not tested	Acceptable	Plos One, 2014, 9: e113345; DNA Res., 2018, 25, 395-407
ORN_306F	ggggccgggggcgcugggcuguccc	human *IRF-1*	25	94	74	Not tested	Acceptable	Plos One, 2014, 9: e113345; DNA Res., 2018, 25, 395-407
ORN-298F	gggcgcugggcuguccc	human *IRF-1*	17	62	59	Not tested	Acceptable	Plos One, 2014, 9: e113345
ORN-310F	ggcuggggccgggggcgcugggcuguccc	human *IRF-1*	29	108	77	Not tested	Acceptable	Plos One, 2014, 9: e113345
ORN-666R	ggccgcugcuggcacagcccc	human *IRF-1*	21	76	66	Not tested	Acceptable	Plos One, 2014, 9: e113345
ORN-363R	cacccuccuggcggggcgggg	human *IRF-1*	21	78	68	Not tested	Acceptable	Plos One, 2014, 9: e113345
ORN-181R	cacccucuccggccgggcgcc	human *IRF-1*	21	78	68	Not tested	Acceptable	Plos One, 2014, 9: e113345
ORN-MCS	agagcggccgccaccgcggug	pBluescript (plasmid)	21	76	66	Not tested	Acceptable	Plos One, 2014, 9: e113345
ORN_20b	cggggucucgacauggucac	human *THYN1*	20	66	58	Not tested	Acceptable	DNA Res., 2018, 25, 395-407
ORN_24b	uccgggguctcgacauggucacgc	human *THYN1*	24	80	64	Not tested	Acceptable	DNA Res., 2018, 25, 395-407
ORN_Target	ccucuuccggggucucgacaugg	human *THYN1*	23	76	62	Not tested	Acceptable	DNA Res., 2018, 25, 395-407
ORN_Gx5	caccuccucuacccgaccccc	human *CDKN2A(p16)*	21	72	62	68–72	Acceptable	DNA Res., 2018, 25, 395-407
ORN_p16	gcggcccggggucggguaga	human *CDKN2A(p16)*	20	72	64	Not tested	Acceptable	DNA Res., 2018, 25, 395-407

(1) Tm was calculated using the formula (a + u) * 2 + (g + c) * 4, where a, u, g and c are the number of A, U, G and C bases, respectively.

(2) Tm was calculated using the formula 64.9 + 41 * (g + c - 16.4) / (a + u + g + c), where a, u, g and c are the number of A, U, G and C bases, respectively.

(2) Tm can be calculated using Oligo Calc (http://biotools.nubic.northwestern.edu/OligoCalc.html).

(1) (2) Temperatures below 68ºC are shown in red.

A

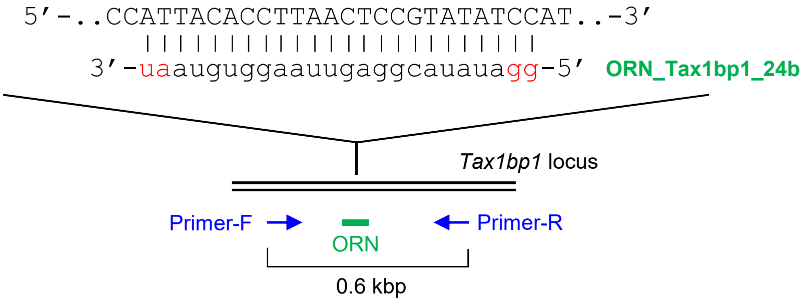

B

gDNA: 20 ng
ORN: 1 μM

Temperature	Time	Cycle
94ºC	2 min	1
98ºC	10 s	30
59, 62 or 65ºC	30 s	
68ºC	50 s	

C

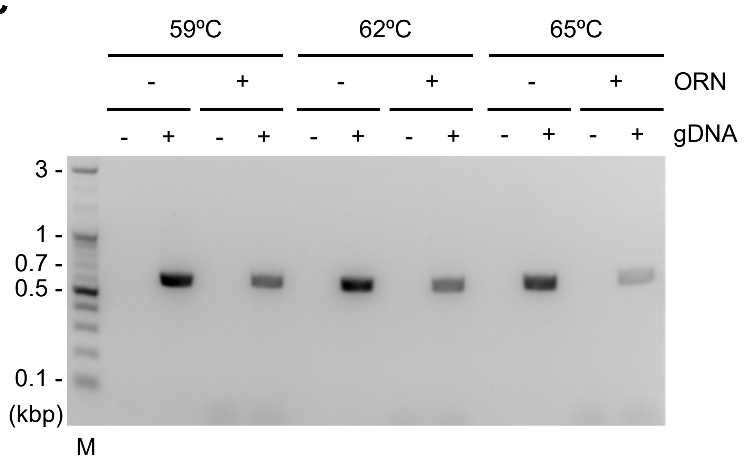

D

gDNA: 20 ng
ORN: 1 μM

Temperature	Time	Cycle
94ºC	2 min	1
98ºC	10 s	30
59, 62 or 65ºC	80 s	

E

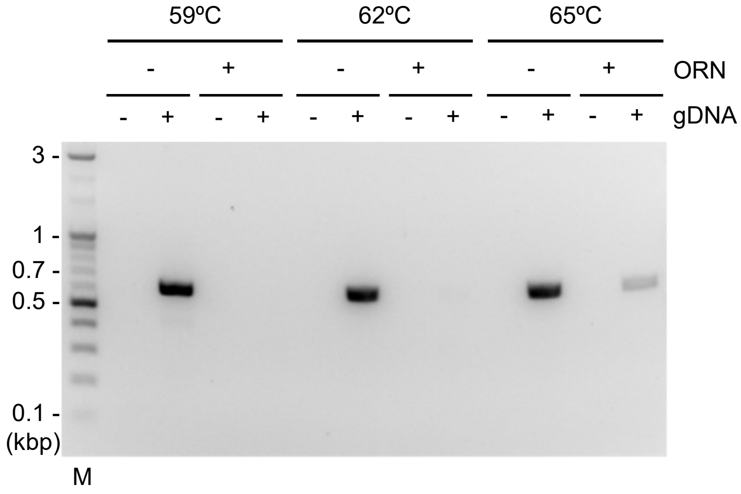

F

Name	Length	Predicted Tm(1)	Predicted Tm(2)	Practical Tm
ORN_Tax1bp1	20 bases	54ºC	46ºC	53 – 56ºC
ORN_Tax1bp1_24b	24 bases	66ºC	52ºC	62 – 65ºC

Supplementary Figure S1

A

gDNA: 20 ng
ORN: 1 μM

Temperature	Time	Cycle
94°C	2 min	1
98°C	10 s	30
50, 56, 62 or 68°C	30 s	
68°C	50 s	

B

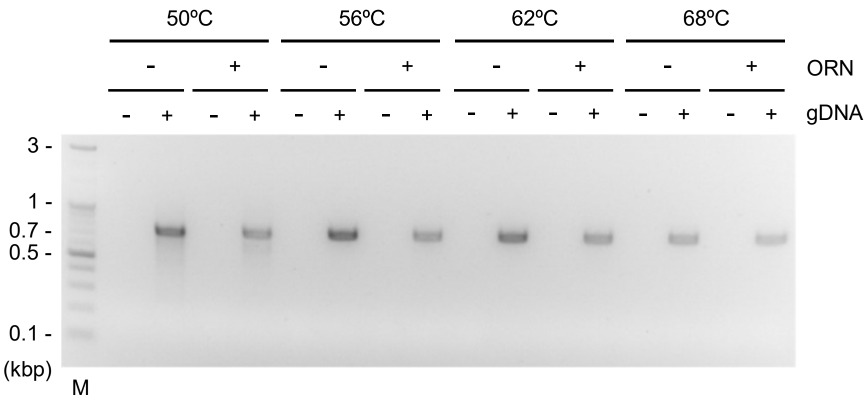

Supplementary Figure S2

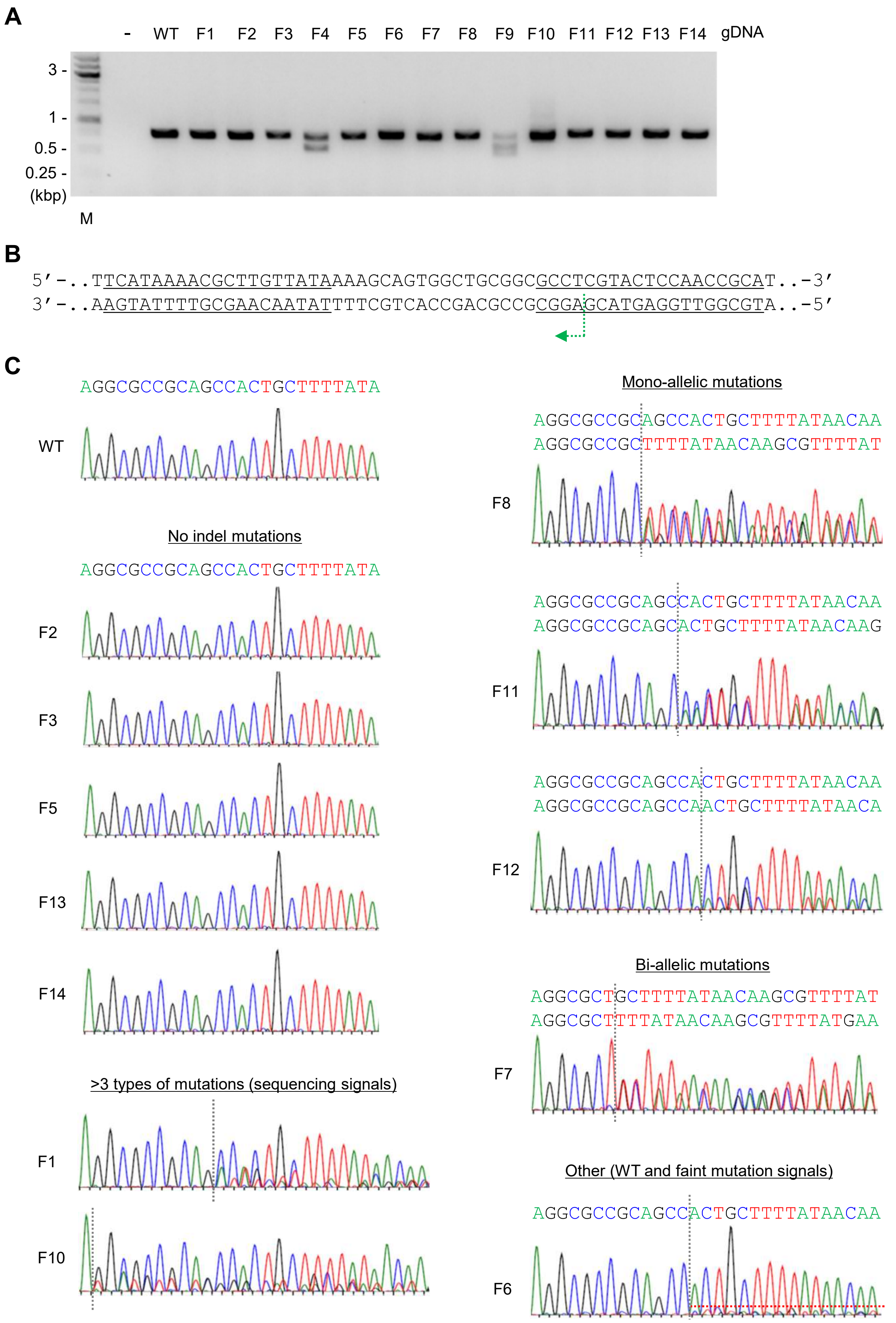

Supplementary Figure S3

```
WT   5'-..TTCATAAAACGCTTGTTATAAAAGCAGTGGCTGCGGCGCCTCGTACTCCAACCGCAT..-3'
     5'-..TTCATAAAACGCTTGTTATAAAAGCAGTGGCTGCGGCGCCTCGTACTCCAACCGCAT..-3'

F7   5'-..TTCATAAAACGCTTGTTATAAAAGCA---------GCGCCTCGTACTCCAACCGCAT..-3'
     5'-..TTCATAAAACGCTTGTTATAAAA------------GCGCCTCGTACTCCAACCGCAT..-3'

F8   5'-..TTCATAAAACGCTTGTTATAAAAGCAGTGGCTGCGGCGCCTCGTACTCCAACCGCAT..-3'
     5'-..TTCATAAAACGCTTGTTATAAAA---------GCGGCGCCTCGTACTCCAACCGCAT..-3'

F11  5'-..TTCATAAAACGCTTGTTATAAAAGCAGTGGCTGCGGCGCCTCGTACTCCAACCGCAT..-3'
     5'-..TTCATAAAACGCTTGTTATAAAAGCAGTG-CTGCGGCGCCTCGTACTCCAACCGCAT..-3'

F12  5'-..TTCATAAAACGCTTGTTATAAAAGCAGTGGCTGCGGCGCCTCGTACTCCAACCGCAT..-3'
     5'-..TTCATAAAACGCTTGTTATAAAAGCAGTGGCTGCGGCGCCTCGTACTCCAACCGCAT..-3'
                                       T
```

Supplementary Figure S4

AGGCGCCGCAGCCACTGCTTTTATAACAA (WT)
AGGCGCCGCAGCACTGCTTTTATAACAAG (1-base deletion) detected

F11

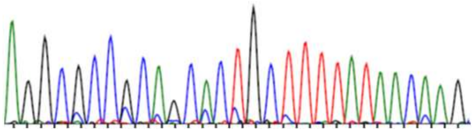

AGGCGCCGCAGCCACTGCTTTTATAACAA (WT)
AGGCGCCGCAGCCAACTGCTTTTATAACA (1-base insertion) detected

F12

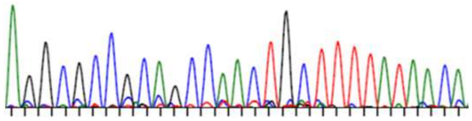

Supplementary Figure S5

A

gDNA: 20 ng
ORN: 1 μM

Temperature	Time	Cycle
94°C	2 min	1
98°C	10 s	30
60°C	80 s	

B

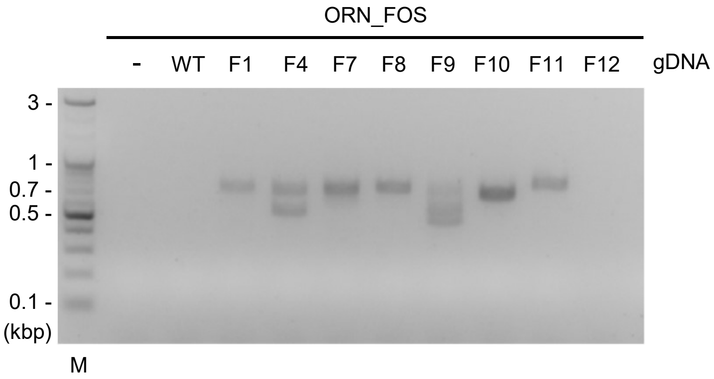

C

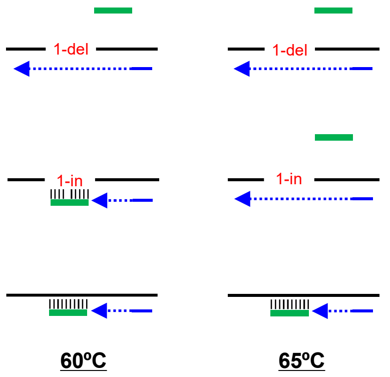

F11: 1-base deletion (1-del) at the target site in one allele

F12: 1-base insertion (1-in) at the target site in one allele

WT: the intact target sequence

D

F11 (RNA bulge)

```
5′-..GTTATAAAAGCAGTGCTGCGGCGCCTCGT..-3′
           |||||||||||||||||||
     3′-uuuucgucacgacgccgcg-5′
                (c)
```

F12 (DNA bulge)

```
               (T)
5′-..GTTATAAAAGCAGTGGCTGCGGCGCCTCGT..-3′
           ||||||||||||||||||||
     3′-uuuucgucaccgacgccgcg-5′
```

Supplementary Figure S6

A

gDNA: 20 ng
ORN: 1 μM

Temperature	Time	Cycle
94°C	2 min	1
98°C	10 s	30
59, 62 or 65°C	80 s	

B

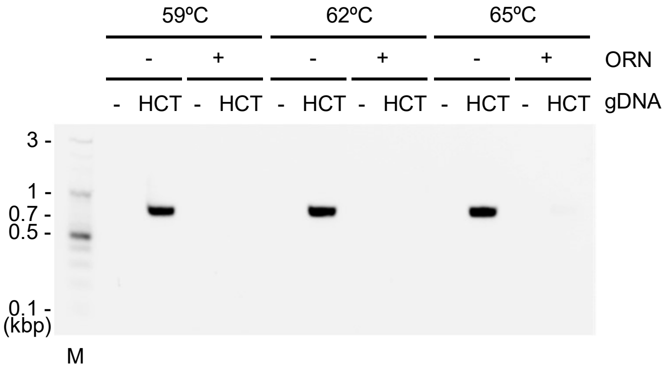

Supplementary Figure S7

A

gDNA: 20 ng
ORN: 0.5 μM

Temperature	Time	Cycle
94°C	2 min	1
98°C	10 s	30
68°C	80 s	

B

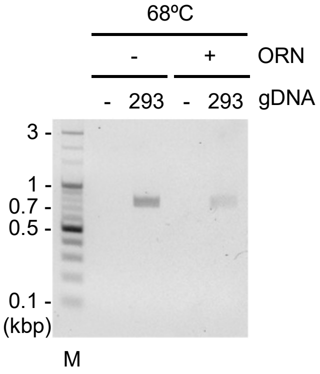

C

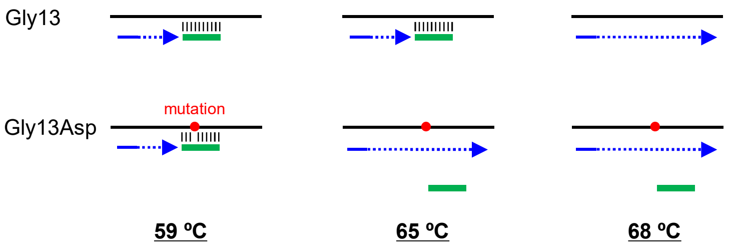

Supplementary Figure S8

A

gDNA: 20 ng
ORN (desalted): 0.5 μM

Temperature	Time	Cycle
94°C	2 min	1
98°C	10 s	30
59, 62 or 65°C	80 s	

B

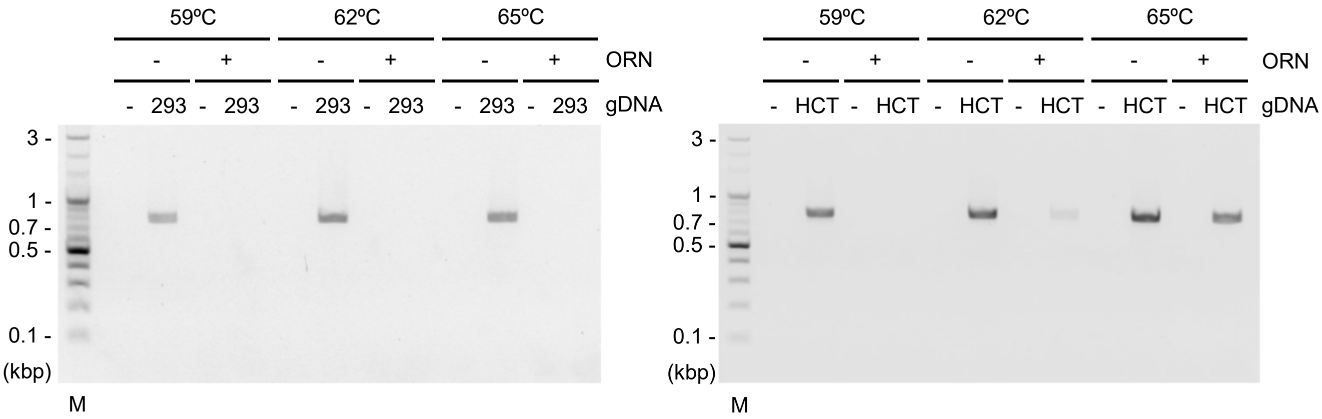

C

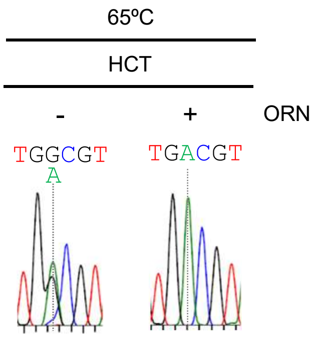

Supplementary Figure S9

A

ORN: 0.25 or 0.5 μM

Temperature	Time	Cycle
94°C	2 min	1
98°C	10 s	30
59 or 68°C	60 s	

B

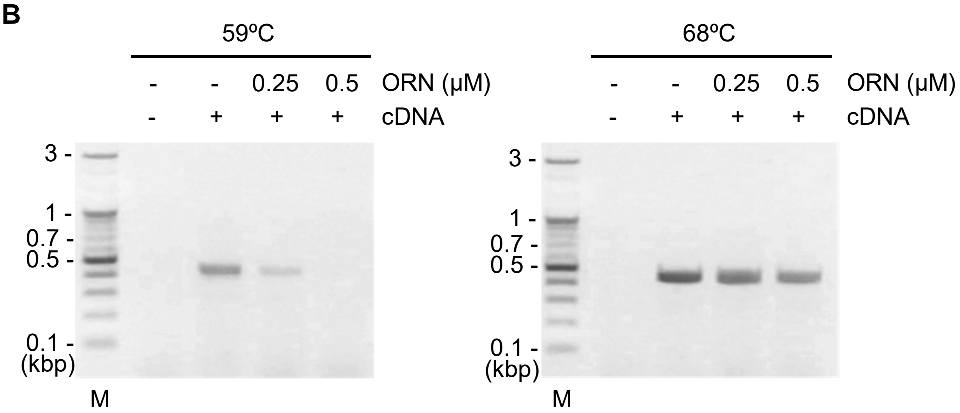

C

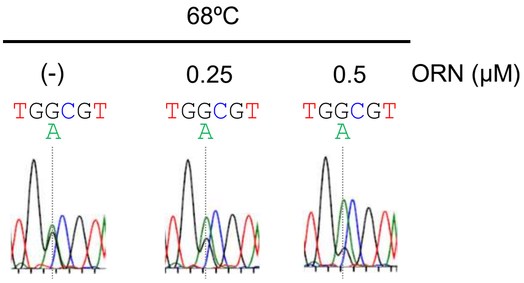

Supplementary Figure S10

A

ORN: 1 μM
Calculated Tm: 64ºC

Temperature	Time	Cycle
94°C	2 min	1
98°C	10 s	35
53, 56, 59, 62 or 65°C	35 s	

B

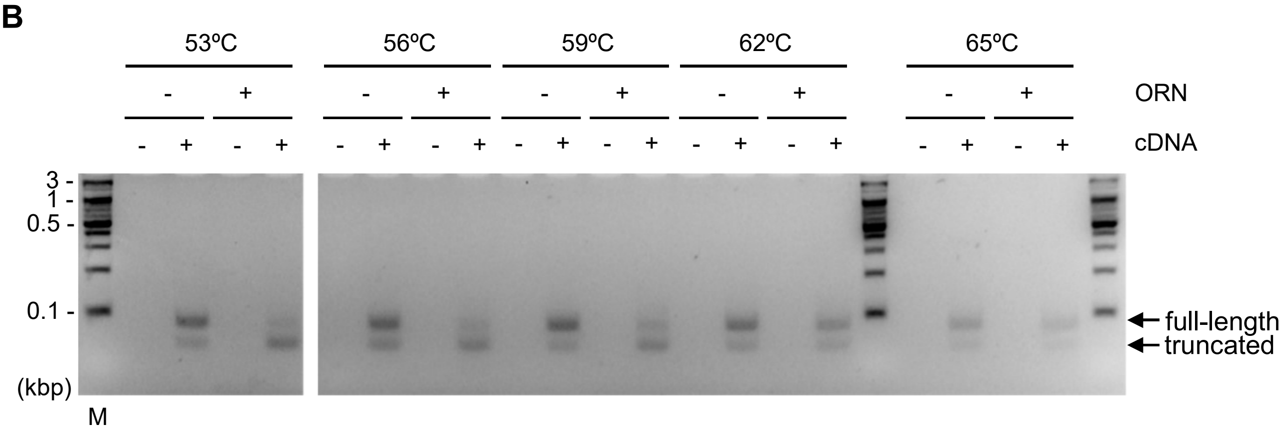

Supplementary Figure S11
